# OTUD5 promotes the growth of hepatocellular carcinoma by deubiquitinating and stabilizing SLC38A1

**DOI:** 10.1186/s13062-024-00475-0

**Published:** 2024-04-24

**Authors:** Yingnan Yang, Siying Jia, Ning Zhu, Xuelian Xiao, Ying Ma, Kangsheng Tu, Yong Guo, Qiuran Xu

**Affiliations:** 1 The Second Clinical Medical College of Zhejiang Chinese Medical University, 310053 Hangzhou, China; 2 Zhejiang Key Laboratory of Tumor Molecular Diagnosis and Individualized Medicine, Zhejiang Provincial People’s Hospital, Affiliated People’s Hospital, Hangzhou Medical College, 310014 Hangzhou, China; 3 Department of Hepatobiliary Surgery, The First Affiliated Hospital of Xi’an Jiaotong University, 710061 Xi’an, China; 4 Department of Cardiovascular Medicine, The Affiliated Hospital of Northwest University, Xi’an No.3 Hospital, 710018 Xi’an, China; 5 Department of Gastrointestinal Surgery, Shangluo Central Hospital, 726000 Shangluo, China

**Keywords:** Hepatocellular carcinoma, Deubiquitinating enzymes, OTUD5, SLC38A1, Tumour growth

## Abstract

**Background:**

Deubiquitinating enzymes (DUBs) cleave ubiquitin on substrate molecules to maintain protein stability. DUBs reportedly participate in the tumorigenesis and tumour progression of hepatocellular carcinoma (HCC). OTU deubiquitinase 5 (OTUD5), a DUB family member, has been recognized as a critical regulator in bladder cancer, breast cancer and HCC. However, the expression and biological function of OTUD5 in HCC are still controversial.

**Results:**

We determined that the expression of OTUD5 was significantly upregulated in HCC tissues. High levels of OTUD5 were also detected in most HCC cell lines. TCGA data analysis demonstrated that high OTUD5 expression indicated poorer overall survival in HCC patients. OTUD5 silencing prominently suppressed HCC cell proliferation, while its overexpression markedly enhanced the proliferation of HCC cells. Mass spectrometry analysis revealed solute carrier family 38 member 1 (SLC38A1) as a candidate downstream target protein of OTUD5. Coimmunoprecipitation analysis confirmed the interaction between OTUD5 and SLC38A1. OTUD5 knockdown reduced and OTUD5 overexpression increased SLC38A1 protein levels in HCC cells. However, OTUD5 alteration had no effect on SLC38A1 mRNA expression. OTUD5 maintained SLC38A1 stability by preventing its ubiquitin-mediated proteasomal degradation. SLC38A1 silencing prominently attenuated the OTUD5-induced increase in HCC cell proliferation. Finally, OTUD5 knockdown markedly suppressed the growth of HCC cells in vivo.

**Conclusions:**

OTUD5 is an oncogene in HCC. OTUD5 contributes to HCC cell proliferation by deubiquitinating and stabilizing SLC38A1. These results may provide a theoretical basis for the development of new anti-HCC drugs.

**Supplementary Information:**

The online version contains supplementary material available at 10.1186/s13062-024-00475-0.

## Background

Many factors affect people’s lives and health, and cancer is one of the most significant factors [1]. The number of patients diagnosed with hepatocellular carcinoma (HCC), which is one of the most common malignant tumours in China, is increasing annually [2]. The onset of HCC is insidious, and most patients present with middle-stage or advanced-stage disease at diagnosis. Early diagnosis in clinical practice is limited because of the complexity the disease and lack of knowledge regarding the underlying mechanism. Moreover, the effect of current therapeutic strategies for HCC is not satisfactory. Identification of accurate tumour markers and therapeutic targets is urgently needed.

Ubiquitination is a common modification of proteins and is ubiquitous in cellular processes [3]. Ubiquitin is attached to substrate molecules via E1 activating enzymes, E2 ubiquitin-conjugating enzymes, and E3 ubiquitin ligases [3]. Deubiquitinating enzymes (DUBs) are able to reverse this process [4]. Approximately 100 DUBs are encoded in the human genome and are divided into seven families based on sequence and structure: ubiquitin carboxyl-terminal hydrolases (UCHs), ubiquitin-specific proteases (USPs), otubain/ovarian tumour domain-containing proteins (OTUs), the Machado-Joseph disease domain (MJD) superfamily, monocyte chemotactic protein-induced proteins (MCPIPs), JAB1/MPN/MOV34 proteases (JAMMs) and the novel motif interacting with ubiquitin-containing DUBs (MINDY) family [4, 5]. Many studies have shown that DUBs are involved in the development and progression of cancer and regulate various biological functions, including cell proliferation and cell cycle-mediated DNA damage repair [6]. In breast cancer, OTUD3 plays a tumour suppressor role by inhibiting the PI3K/AKT signalling pathway through the deubiquitination and stabilization of PTEN, thereby inhibiting tumour progression [7]. OTUD7B functions as an inhibitor of the noncanonical NF-κB pathway by deubiquitinating TNF receptor-associated Factor 3 (TRAF3) [8]. OTUB2 promotes the progression of breast cancer by directly deubiquitinating and stabilizing YAP and TAZ to activate the YAP signalling pathway [9]. OTUD5 is a member of the OTU family. Recently, studies have shown that OTUD5 plays an indispensable role in cancer [10–13]. A recent study revealed that OTUD5 can be phosphorylated by mTOR and activate its catalytic activity and is positively correlated with the mTOR signalling pathway [13]. OTUD5 can activate the mTOR signalling pathway by deubiquitinating ring finger protein 186 (RNF186), thereby promoting the progression of bladder cancer [10]. In breast cancer, OTUD5 deubiquitinates and stabilizes YAP in macrophages, and YAP promotes M2 macrophage polarization, which is beneficial for enhancing the metastatic ability of breast cancer cells [12]. A recent study reported that OTUD5 expression is strongly downregulated in HCC and that its knockdown accelerates cancer cell growth both in vitro and in vivo [11]. However, analysis of The Cancer Genome Atlas (TCGA) and Clinical Proteomic Tumour Analysis Consortium (CPTAC) data from UALCAN (https://ualcan.path.uab.edu/analysis.html) showed that OTUD5 is highly expressed in HCC and that its overexpression predicts a poor patient prognosis, indicating that the expression of OTUD5 in HCC remains controversial.

In this study, we investigated the expression of OTUD5 in HCC. We verified the effect of OTUD5 on the proliferation of HCC cells in vitro and in vivo. Finally, the interaction between OTUD5 and solute carrier family 38 member 1 (SLC38A1) and the underlying regulatory mechanism were investigated. Our results suggest that OTUD5 is an oncogene in HCC that controls the growth of cancer cells by deubiquitinating and stabilizing SLC38A1.

## Methods

### Clinical specimens

Forty-four pairs of cancer and matched paracancerous tissues were obtained from HCC patients at the First Affiliated Hospital of Xi’an Jiaotong University with the consent of the patients. None of the patients received any preoperative treatment. All tissues were confirmed by postoperative pathology and stored at -80 °C. Patients with other malignant tumours were excluded from this study. The Research Ethics Committee of The First Affiliated Hospital of Xi’an Jiaotong University approved the research protocol of this study (No: XJTU1AF2020LSY-08).

### Cell culture

Human embryonic kidney cells (HEK293T), normal liver cells (MIHA) and HCC cell lines (HepG2, Hep3B, Huh7, HCCLM3, PLC/PRF/5, and MHCC97L) were maintained in our laboratory [14, 15]. Mycoplasma contamination was regularly detected using a Mycoplasma contamination detection kit (InvivoGen, San Diego, CA, USA). All cells were cultured in Dulbecco’s modified Eagle’s medium (DMEM) (Gibco; Thermo Fisher Scientific, Waltham, MA, USA) supplemented with 10% foetal bovine serum (FBS) (ExCell Bio, Shanghai, China) and incubated at 37 °C in 5% CO_2_.

### Cell transduction and lentivirus packaging

OTUD5 shRNAs (shOTUD5-1 and shOTUD5-2), SLC38A1 shRNA (shSLC38A1), nontargeting shRNA (NT shRNA), Flag-OTUD5 and empty vector were purchased from Shanghai Genechem Co., Ltd. Cell transduction was performed with Lipofectamine™ 3000 (Invitrogen; Thermo Fisher Scientific). A six-well plate was seeded with cells, and the transduction operation was performed when the cell density reached 50–70% the next day. The medium in the six-well plate was replaced, and the Lipofectamine 3000-p3000-plasmid DNA mixture was prepared and added to the six-well plate. The cells were cultured for 48 h before the transduction efficiency was analysed. Lentivirus packaging was performed in HEK293T cells using psPAX2, pMD2.G and corresponding plasmids according to previously described protocols [16]. Polybrene (8 µg/ml, Beyotime, Shanghai, China) was used to increase the efficiency of lentivirus infection. The shRNA sequences used are listed in Supplementary Table 1.

### Western blotting (WB)

The samples were lysed on ice using RIPA lysis buffer (Beyotime) for 30 min and centrifuged at 4 °C for 20 min. The supernatant was extracted and quantified using a BCA protein assay kit (Beyotime). After determining the desired amount of protein, 5× loading buffer (Beyotime) was added, and the sample was then deformed by heating in a metal bath for 10 min. Proteins were separated by SDS‒PAGE and transferred to PVDF membranes (Merck Millipore, Darmstadt, Germany). After blocking with 5% skim milk for one hour, the membranes were incubated with the corresponding primary antibodies overnight. The next day, the membranes were washed three times with TBST buffer, and the corresponding secondary antibodies (Beyotime) were then added. The bands were visualized with enhanced chemiluminescence (ECL) reagent (Merck Millipore) and imaged with a luminescence visualizer. The grey values were analysed with ImageJ software (NIH, Bethesda, MD, USA). MG132 (MCE, Monmouth Junction, NJ, USA) was used to block proteasomal degradation, and cycloheximide (CHX, MEC) was used to inhibit protein synthesis. The following antibodies were used: anti-OTUD5 (#20,087, Cell Signaling Technology, Danvers, MA, USA), anti-SLC38A1 (#36,057, Cell Signaling Technology), and anti-GAPDH (60004-1-Ig, Proteintech, Wuhan, China).

### Quantitative reverse transcription polymerase chain reaction (RT‒qPCR)

RT‒qPCR was performed according to the manufacturer’s protocols. RNA isolation, reverse transcription and PCR amplification were carried out with TRIzol reagent (Invitrogen; Thermo Fisher Scientific), the RevertAid First Strand cDNA Synthesis Kit (Thermo Fisher Scientific) and 2× Universal SYBR Green Fast qPCR Mix (ABclonal, Woburn, MA), respectively. The following primers were purchased from Tsingke Biotech (Beijing, China).

 OTUD5 F: 5’-CCCATCAACACATTCCATG-3’

 OTUD5 R: 5’-CATCAGAGACTGCTCTGCAAA-3’

 SLC38A1 F: 5’-GCTTTGGTTAAAGAGCGGGC-3’

 SLC38A1 R: 5’-CTGAGGGTCACGAATCGGAG-3’

### Coimmunoprecipitation (Co-IP) analysis

Co-IP assays were performed using the Dynabeads™ Protein G Immunoprecipitation Kit (Invitrogen; Thermo Fisher Scientific). The magnetic beads were first bound to the corresponding antibody. Cells were lysed with IP lysis buffer, and the resulting protein samples were added to the antibody-magnetic bead mixture and incubated overnight at 4 °C. The beads were washed three times with cleaning solution. Then, 20 µL of eluate and 5 µL of 5× loading buffer were added to the beads, which were denatured by heating at 70 °C for 10 min. The magnetic beads were removed with a magnetic rack. Immunoblotting was then performed. An anti-OTUD5 rabbit antibody (#20,087, Cell Signaling Technology) was used for immunoprecipitation, and an anti-SLC38A1 mouse antibody (sc-137,032, Santa Cruz Biotechnology, Dallas, Texas, USA) was used for immunoblotting. An anti-SLC38A1 mouse antibody (sc-137,032, Santa Cruz Biotechnology) was used for immunoprecipitation, and an anti-OTUD5 rabbit antibody (#20,087, Cell Signaling Technology) and an anti-Ub rabbit antibody (#43,124, Cell Signaling Technology) were used for immunoblotting.

### Mass spectrometry analysis

The protein was isolated from HCCLM3 cells with or without OTUD5 knockdown and subjected to mass spectrometry analysis (MetWare Biotechnology, Wuhan, China).

### Cell counting Kit-8 (CCK-8) assay

A CCK-8 kit (Beyotime) was used for detection. A total of 2 × 10^3^ cells were incubated in 96-well plates and then attached as a starting point for testing. After 24, 48 and 72 h of incubation, 100 µL of CCK-8/DMEM mixture (1:10) was added to each well and incubated at 37 °C for 2 h. The absorbance at a wavelength of 450 nm was measured with an enzyme label instrument, and the results were analysed.

### 5-Ethynyl-2’-deoxyuridine (EdU) assay

The Cell-Light EdU In Vitro Kit (RiboBio, Guangzhou, China) was used. The cells were plated in 24-well plates at a moderate density. After the addition of 10 µM EdU reagent and incubation for 2 h at 37 °C, the cells were fixed with 4% paraformaldehyde for 30 min, and images were acquired via fluorescence microscopy.

### Immunohistochemistry (IHC)

IHC was performed with a commercial kit (ZSGB-BIO, Beijing, China) following the manufacturer’s instructions. The IHC score was calculated as previously described [16].

### Animal experiments

HCCLM3 cells with OTUD5 knockdown or control cells (5 × 10^6^ per injection) were injected into the backs of 4-week-old BALB/C male nude mice. The long and short diameters of the tumours were measured every four days, and the tumour volume was calculated according to the following formula: V = length × width × width/2. Four weeks after implantation, the mice were sacrificed after carbon dioxide inhalation, and the tumours were collected for weight measurement and IHC staining of OTUD5 and SLC38A1. All animal experiments were approved by the Ethics Review Committee of Xi’an Jiaotong University (No. XJTUAE2023-278).

### Statistical analysis

Statistical analysis was performed using GraphPad Prism software (GraphPad Inc., San Diego, CA, USA) in this study. The experimental data are presented as the mean ± standard deviation (SD) of three independent repeated experiments. Differences between two groups were compared by t tests, and differences among multiple groups were analysed by ANOVA. *P* < 0.05 was considered to indicate statistical significance.

## Results

### OTUD5 overexpression predicts an unfavourable prognosis in HCC patients

**Fig. 1:**
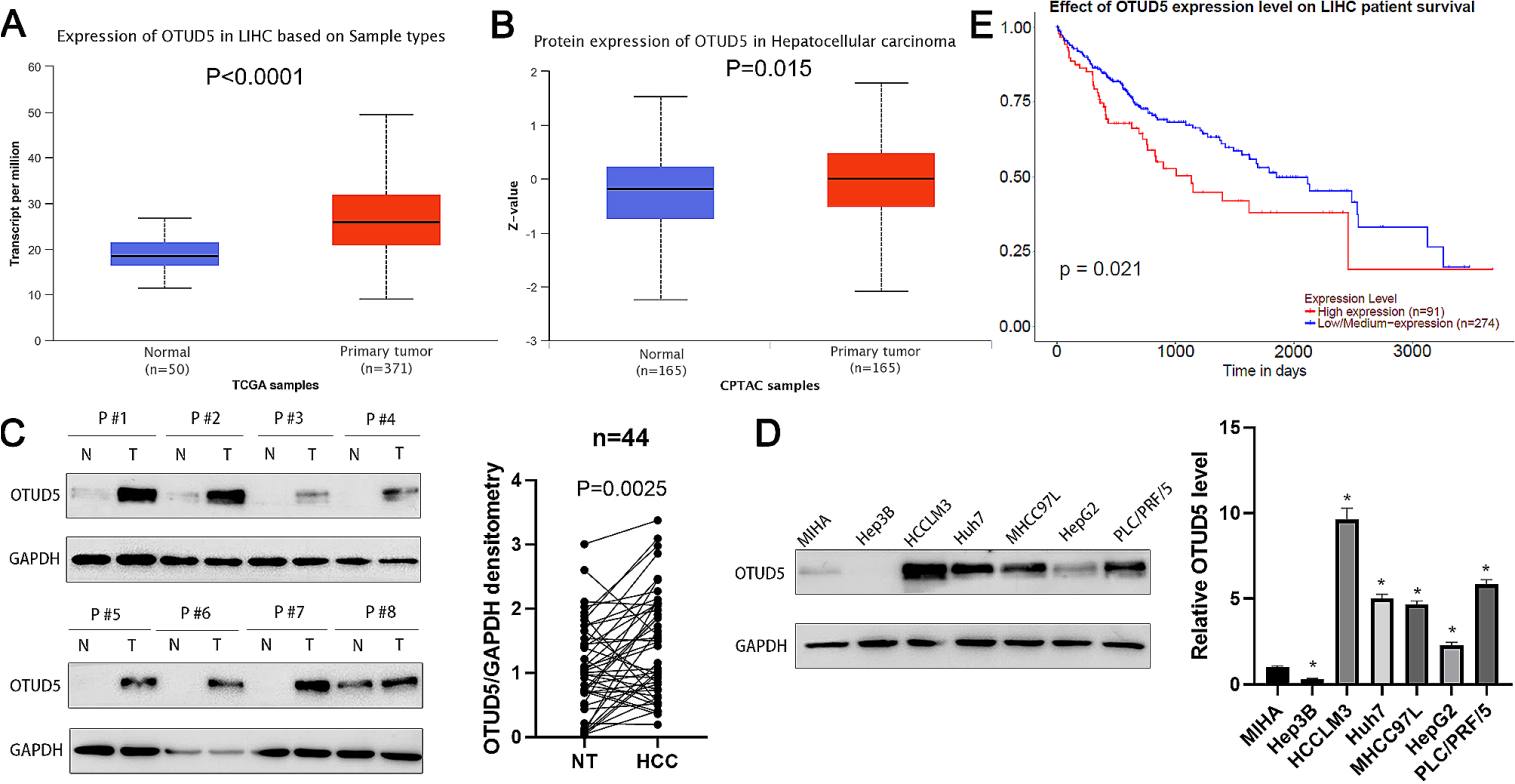
The expression of OTUD5 and survival analysis based on OTUD5 expression in HCC. (**A**) Differences in the expression of OTUD5 mRNA in HCC and normal liver tissues based on TCGA-LIHC data. (**B**) Differences in the expression of the OTUD5 protein in HCC and normal liver tissues based on CPTAC data. (**C**) Western blotting was used to detect the expression of OTUD5 in forty-four pairs of HCC tissues and corresponding tumour-adjacent tissues. (**D**) Western blotting was used to detect the expression of OTUD5 in MIHA cells and a variety of HCC cell lines. (**E**) The correlation between the OTUD5 expression level and HCC patient survival was analysed. *P < 0.05

According to the UALCAN platform (https://ualcan.path.uab.edu/analysis.html), TCGA data showed that the expression of OTUD5 mRNA in HCC tissues was prominently greater than that in normal liver tissues (*P* < 0.0001, Fig. 1A), and CPTAC data further confirmed the upregulation of the OTUD5 protein in HCC tissues compared to that in normal tissues (*P* = 0.015, Fig. 1B). Next, forty-four pairs of HCC and tumour-adjacent tissues were harvested for RT‒qPCR and WB. Our results indicated that OTUD5 was frequently overexpressed in HCC tissues (*P* < 0.05, Fig. 1C and Supplementary Fig. 1). Compared with those in normal liver cells (MIHA), the levels of OTUD5 were markedly greater in HCCLM3, Huh7, MHCC97L, HepG2 and PLC/PRF/5 cells (*P* < 0.05, Fig. 1D). Importantly, HCC patients with high OTUD5 expression had a worse overall survival rate than did those with low/medium OTUD5 expression (*P* = 0.021, Fig. 1E). These data suggest that OTUD5 is a potential oncogene in HCC.

### OTUD5 promotes the proliferation of HCC cells

**Fig. 2:**
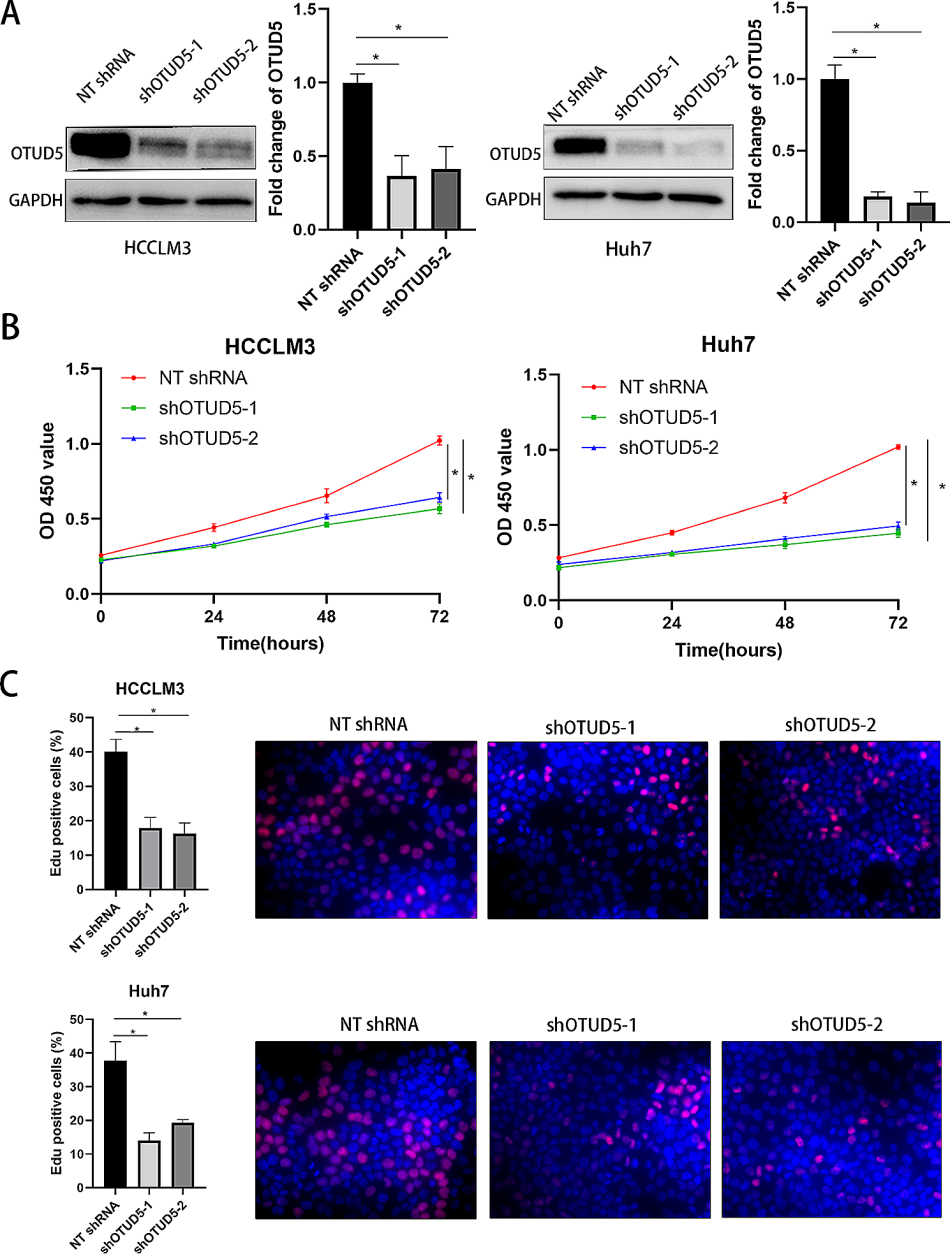
TUD5 knockdown inhibits HCC cell proliferation. (**A**) OTUD5 shRNAs (shOTUD5-1 and shOTUD5-2) and NT shRNA were transduced into HCCLM3 and Huh7 cells, respectively, and the knockdown efficiency was verified by Western blotting. (**B**) A CCK-8 assay was used to investigate the effect of OTUD5 knockdown on the viability of HCC cells. (**C**) An EdU assay was used to detect the proliferation of HCC cells in the OTUD5 knockdown and control groups. *P < 0.05

**Fig. 3:**
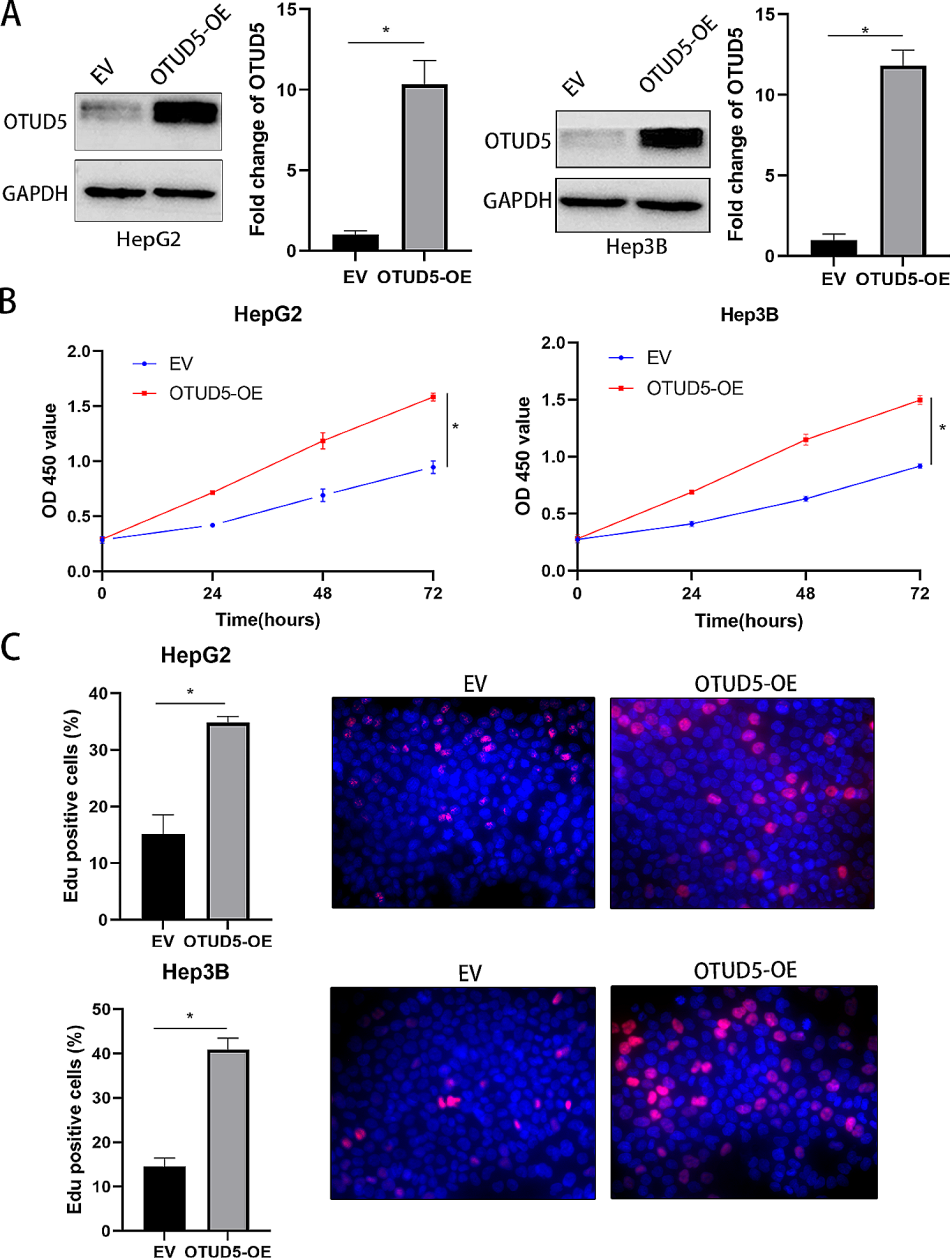
OTUD5 overexpression promotes HCC cell proliferation. (**A**) Plasmids overexpressing OTUD5 were transduced into HepG2 and Hep3B cells, and the transduction efficiency was verified by Western blotting. (**B**) A CCK-8 assay was used to investigate the effect of OTUD5 overexpression on the viability of HCC cells. (**C**) An EdU assay was used to detect the proliferation of HCC cells in the OTUD5-overexpressing and control groups. *P < 0.05

**Fig. 4:**
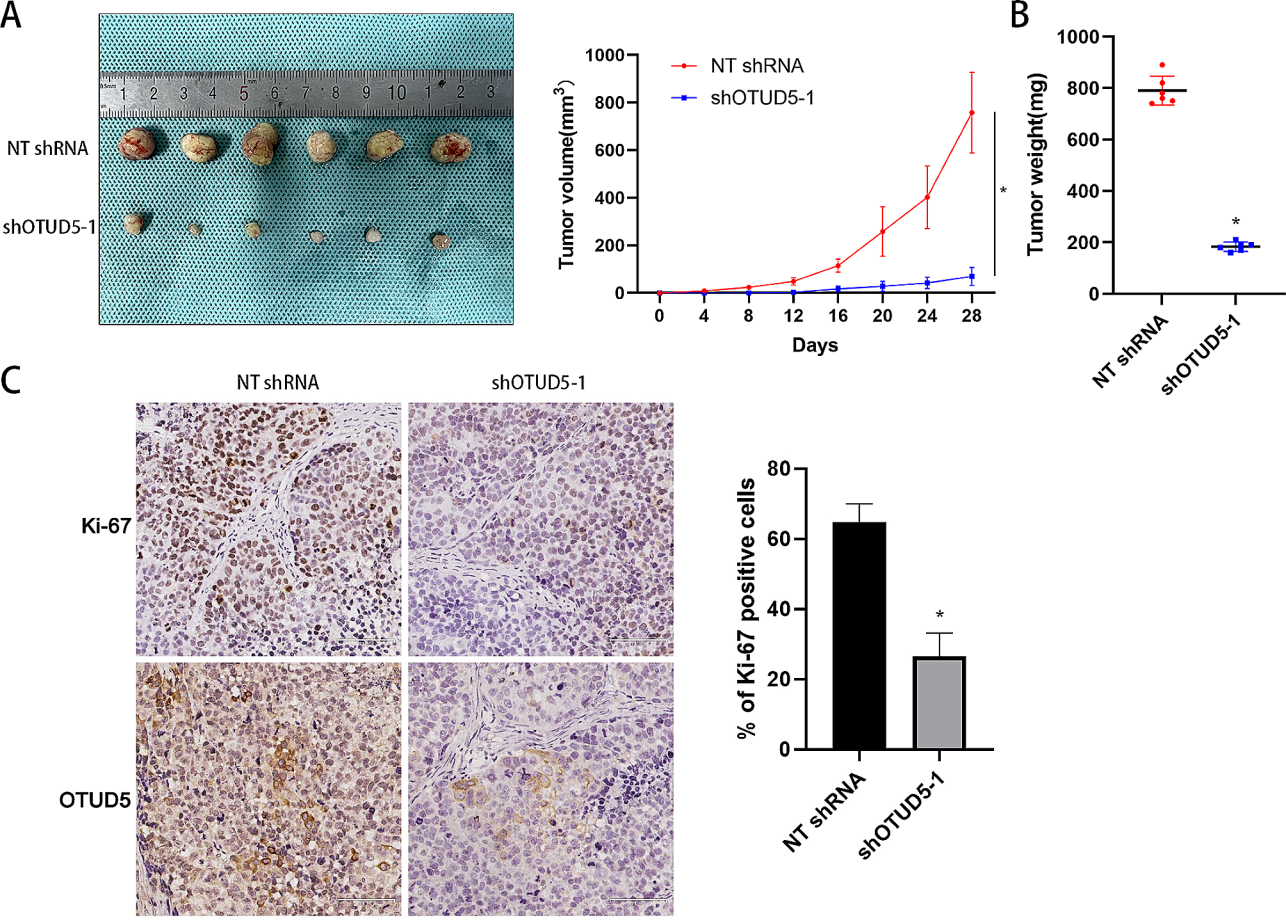
OTUD5 knockdown suppresses the growth of HCC cellsin vivo. (**A**) HCCLM3 cells with OTUD5 knockdown or control cells were injected subcutaneously into the flanks of nude mice. OTUD5 knockdown attenuated tumour growth in vivo. (**B**) The tumour weight in the OTUD5 knockdown group was significantly lower than that in the control group. (**C**) IHC staining was used to detect the expression of OTUD5 and Ki-67 in subcutaneous tumours of the OTUD5 knockdown group and control group. *P < 0.05

Subsequently, we detected whether OTUD5 regulates the proliferation of HCC cells. OTUD5 expression was dramatically reduced after lentiviral transduction of shRNAs into HCCLM3 and Huh7 cells (*P* < 0.05, Fig. 2A). CCK-8 assays verified that OTUD5 silencing decreased the viability of HCC cells (*P* < 0.05, Fig. 2B). According to the results of the EdU assays, cell proliferation was strongly inhibited by OTUD5 knockdown in HCC cells (*P* < 0.05, Fig. 2C). In addition, OTUD5 was overexpressed in HepG2 and Hep3B cells (*P* < 0.05, Fig. 3A). Ectopic expression of OTUD5 resulted in a significant increase in HCC cell proliferation (*P* < 0.05, Fig. 3B and C). We further explored the function of OTUD5 in vivo. OTUD5 knockdown significantly inhibited the growth of HCCLM3 cells in nude mice, as determined by the significant reductions in tumour volume and weight (*P* < 0.05, Fig. 4A and B). OTUD5 knockdown significantly reduced the IHC score of Ki-67 in xenograft tumour tissues (*P* < 0.05, Fig. 4C). Therefore, OTUD5 is involved in the regulation of HCC cell growth.

### OTUD5 binds to SLC38A1

**Fig. 5:**
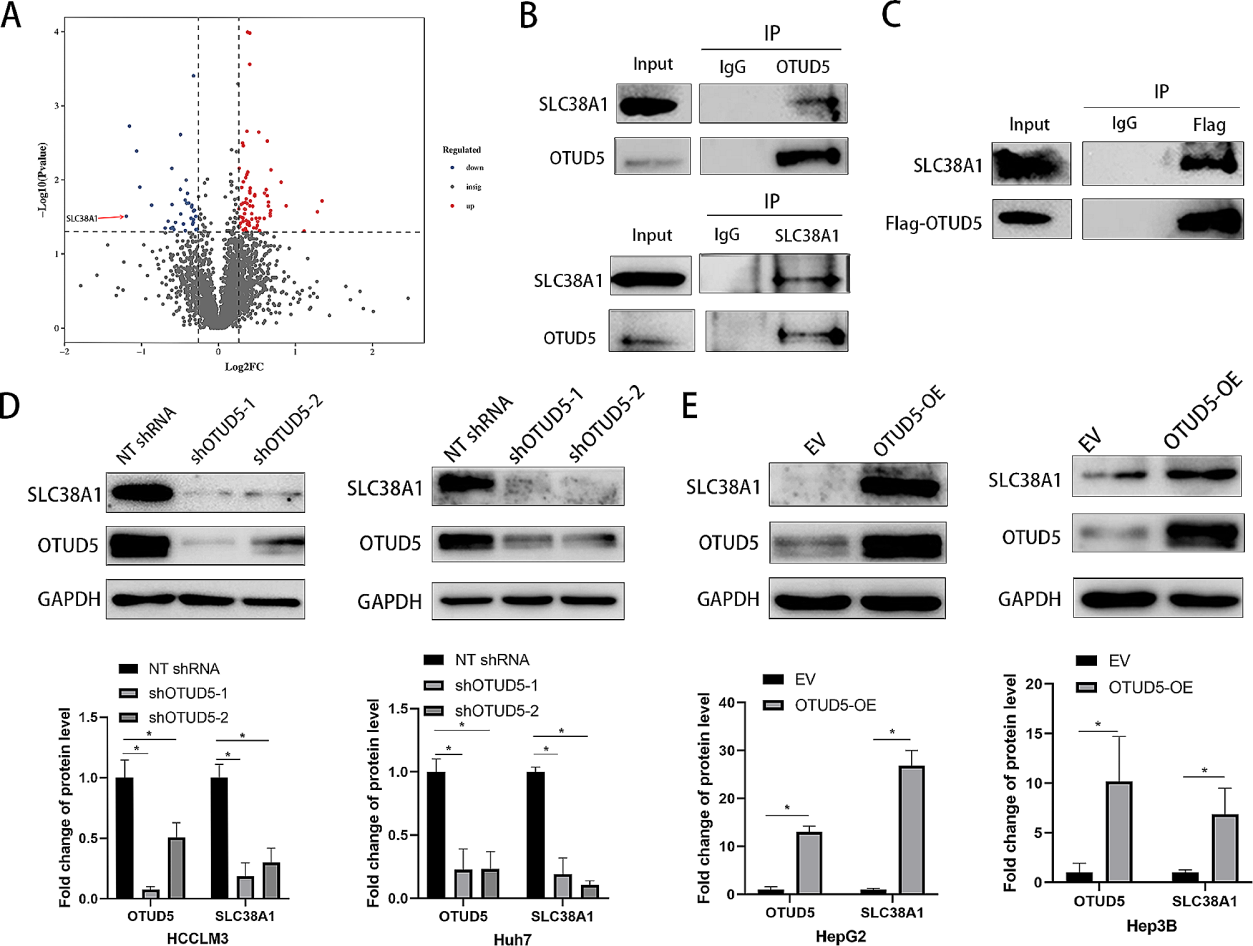
OTUD5 binds to SLC38A1. (**A**) Label-free mass spectrometry was performed on HCCLM3 cells with or without OTUD5 knockdown to identify differentially expressed proteins. (**B**) The binding between endogenous OTUD5 and SLC38A1 in HCCLM3 cells was demonstrated by co-IP. (**C**) The flag-tagged OTUD5 plasmid was transduced into HEK293T cells. Proteins were precipitated with a FLAG-tagged antibody, and SLC38A1 was detected by Western blotting. (**D**) HCCLM3 and Huh-7 cells that were transduced with OTUD5 shRNAs or NT shRNA were subjected to Western blotting for SLC38A1 and OTUD5 expression. (**E**) HepG2 and Hep3B cells that were transduced with an empty vector (EV) or an OTUD5 expression vector were subjected to Western blotting for SLC38A1 and OTUD5 expression. *P < 0.05

To thoroughly investigate the regulatory mechanisms of OTUD5 in HCC, the differences in the expression of proteins in HCCLM3 cells with or without OTUD5 knockdown were analysed via label-free quantitative proteomics technology. Thirty-six proteins were downregulated, and 69 proteins were upregulated by OTUD5 knockdown. The SLC38A1 protein was the most strongly downregulated by OTUD5 knockdown (Fig. 5A and Supplementary Table 2). Co-IP assays confirmed that both exogenous and endogenous OTUD5 bound to SLC38A1 (Fig. 5B and C). Next, we determined the expression of SLC38A1 in HCC cells with altered OTUD5 expression. The results showed that SLC38A1 protein expression was markedly decreased by OTUD5 knockdown (*P* < 0.05, Fig. 5D). We subsequently found that the protein level of SLC38A1 was increased by OTUD5 overexpression (Fig. 5E). OTUD5 knockdown significantly reduced the IHC score of SLC38A1 in xenograft tumour tissues (*P* < 0.05, Supplementary Fig. 2). However, we demonstrated that overexpression or knockdown of OTUD5 did not affect the mRNA levels of SLC38A1 (Supplementary Fig. 3). Thus, our results suggest that OTUD5 is a novel regulator of SLC38A1 in HCC cells.

### OTUD5 regulates the deubiquitination and stability of SLC38A1

**Fig. 6:**
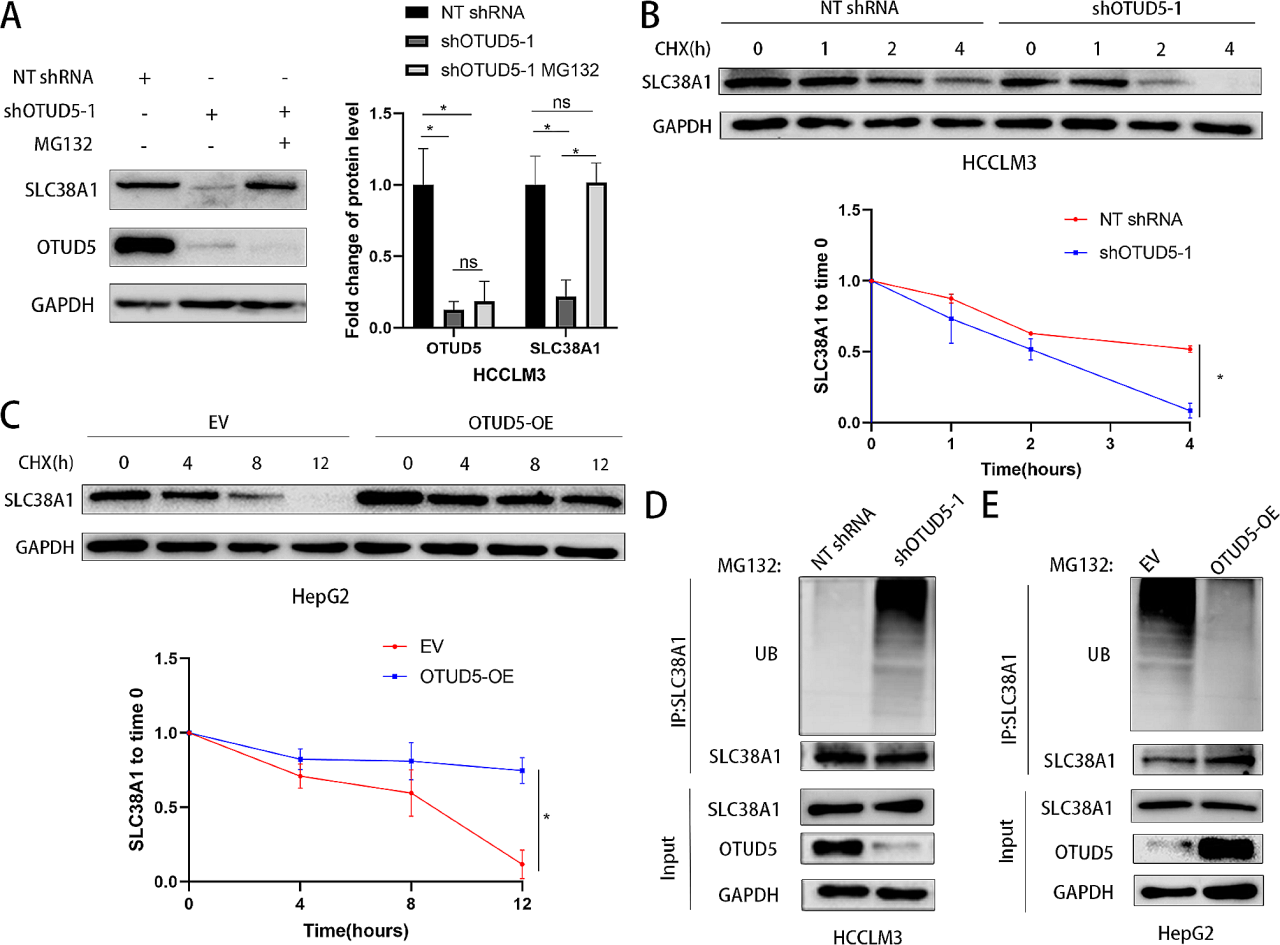
OTUD5 deubiquitinates and stabilizes SLC38A1 in HCC cells. (**A**) HCCLM3 cells with OTUD5 knockdown were treated with or without MG132. The downregulation of SLC38A1 protein induced by OTUD5 knockdown was reversed by MG132. (**B** and **C**) OTUD5 knockdown shortened and OTUD5 overexpression prolonged the half-life of the SLC38A1 protein in HCC cells. (**D** and **E**) OTUD5 knockdown promoted but OTUD5 overexpression suppressed the ubiquitination of OTUD5 in HCC cells. *P < 0.05

To determine whether the reduction in SLC38A1 expression induced by OTUD5 knockdown is dependent on the proteasomal degradation pathway, MG132 was used to block proteasome activity. Our results indicated that MG132 treatment markedly reversed the OTUD5 knockdown-induced SLC38A1 downregulation in HCC cells (*P* < 0.05, Fig. 6A and Supplementary Fig. 4A). Moreover, CHX was used to block protein synthesis. We found that OTUD5 knockdown led to faster SLC38A1 degradation and that OTUD5 overexpression prolonged the half-life of SLC38A1 in HCC cells (*P* < 0.05, Fig. 6B and C and Supplementary Fig. 4B and 4 C). Next, we determined whether OTUD5 prevents the ubiquitination of SLC38A1. The results showed that the knockdown of OTUD5 increased the level of ubiquitinated SLC38A1 in HCC cells (Fig. 6D and Supplementary Fig. 4D), while the level of ubiquitinated SLC38A1 was significantly reduced in HCC cells overexpressing OTUD5 (Fig. 6E and Supplementary Fig. 4E). Therefore, these results support that OTUD5 deubiquitinates and stabilizes SLC38A1 in HCC.

### SLC38A1 mediates the effect of OTUD5 on HCC

**Fig. 7:**
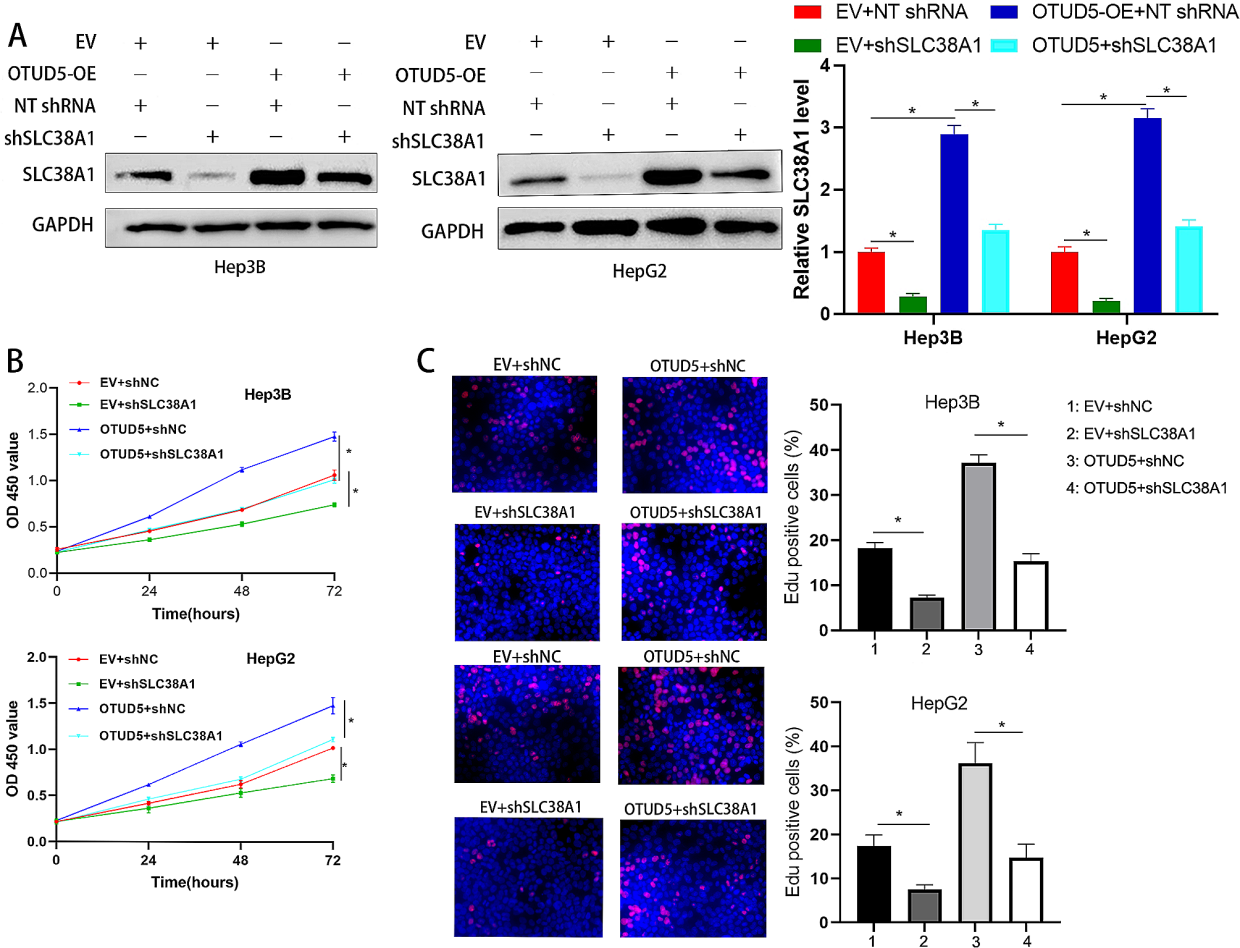
SLC38A1 knockdown abolishes OTUD5-enhanced HCC cell proliferation. (**A**) Hep3B and HepG2 cells that were transduced with the corresponding vectors were subjected to Western blotting for SLC38A1 expression. (**B** and **C**) OTUD5 overexpression led to enhanced HCC cell proliferation, which was strongly reversed by SLC38A1 knockdown. *P < 0.05

To further confirm whether OTUD5-induced cell growth involves SLC38A1, we knocked down SLC38A1 expression in HCC cells overexpressing OTUD5 (*P* < 0.05, Fig. 7A). We demonstrated that SLC38A1 knockdown significantly decreased the viability and proliferation of Hep3B and HepG2 cells (*P* < 0.05, Fig. 7B and C). Importantly, the OTUD5 overexpression-induced increase in HCC cell proliferation was strongly attenuated by SLC38A1 knockdown (*P* < 0.05, Fig. 7B and C). Taken together, these findings indicate that OTUD5 promotes HCC cell proliferation via SLC38A1.

## Discussion

DUBs have a wide range of functions in regulating various cancer-related processes [17]. Notably, the expression pattern of OTUD5 in tumours clearly depends on the tissue type. OTUD5 is poorly expressed in HCC [11] and non-small cell lung cancer (NSCLC) [18, 19], while its overexpression is observed in bladder cancer [10]. In this study, we found that OTUD5 mRNA was highly expressed in HCC and closely associated with a poor prognosis based on data from public databases. The upregulated expression of OTUD5 was also confirmed in our HCC tissues and cell lines. This controversial expression pattern of OTUD5 in HCC caught our attention. A recent study revealed that OTUD5 is upregulated in HepG2 cells after HBV infection and, in turn, facilitates HBV replication [20]. HBV infection and the resulting liver fibrosis and cirrhosis are the main causative factors of HCC in China [21]. Thus, the above evidence seems to support the high expression of OTUD5 in HCC, especially in HBV-related HCC.

In our study, overexpression of OTUD5 promoted the proliferation of HCC cells, while OTUD5 knockdown inhibited cell proliferation, which was also confirmed in vivo. However, another group reported a tumour-suppressive role of OTUD5 in HCC [11]. OTUD5 often functions by regulating the deubiquitination of substrate proteins. OTUD5 acts as a negative regulator of innate immunity to degrade interferon by deubiquitinating and stabilizing TNF receptor-associated factor 3 (TRAF3) [22]. In bladder cancer, OTUD5 activates mechanistic target of rapamycin complex 1 (mTORC1) signalling by deubiquitinating and stabilizing ring finger protein 186 (RNF186) and promoting sestrin2 degradation [10]. OTUD5 targets β-transducin repeat-containing protein 1 (β-TrCP1) for deubiquitination to stabilize DEP domain containing MTOR interacting protein (DEPTOR), thereby promoting the activation of mTOR signalling [13]. OTUD5-induced deubiquitination of tripartite motif containing 25 (TRIM25) increases transcriptional activity but not protein stability [11]. To reveal the mechanism underlying the tumour-promoting role of OTUD5 in HCC, we screened the potential substrate proteins of OTUD5 using IP-MS analysis. SLC38A1, the most downregulated protein after OTUD5 knockdown, was found to be a candidate substrate of OTUD5. SLC38A1 is a glutamine transporter. Glutamine plays an essential role in essential amino acid uptake and maintenance of mTOR signalling pathway activation [23]. SLC38A1 is highly expressed in breast cancer, HCC, gastric cancer, and colorectal cancer and is associated with proliferation, metastasis, invasion, cell death, and poor prognosis [24–29]. SLC38A1 is integral for YAP regulation of mTORC1; SLC38A1 is required for YAP1/TAZ-mediated mTORC1 activation and is critical for tumour formation, growth and progression in HCC [29]. In HCC, YAP and TAZ regulate the first rate-limiting step of amino acid metabolism, and YAP regulates glutamine uptake through SLC38A1, which directly regulates mTORC1 activity. SLC38A1 silencing attenuated the PI3K/AKT/mTOR pathway and repressed the proliferation and migration of Huh-7 and Hep3B cells [26]. Our further experiments verified the interaction between OTUD5 and SLC38A1. We confirmed that OTUD5 targets SLC38A1 for deubiquitination to block its proteasomal degradation, thereby maintaining SLC38A1 stability. The inhibitory effect of SLC38A1 knockdown on HCC cell proliferation was confirmed in our study. Importantly, SLC38A1 knockdown significantly attenuated the OTUD5-induced increase in proliferation of HCC cells. Therefore, our results suggest that OTUD5 promotes HCC cell proliferation by increasing the stability of SLC38A1.

## Conclusions

Our study revealed the upregulation of OTUD5 in HCC. OTUD5 acts as an oncogene by promoting HCC cell proliferation in vitro and in vivo. Importantly, we demonstrated that SLC38A1 is a novel substrate protein of OTUD5 in HCC. SLC38A1 is deubiquitinated and stabilized by OTUD5 and mediates the tumour-promoting role of OTUD5 in HCC. This study provides new insights into the function of OTUD5 and the regulatory mechanism of SLC38A1 in HCC.

### Electronic supplementary material

Below is the link to the electronic supplementary material.

Supplementary Material 1

Supplementary Material 2

Supplementary Figure 1 The expression of OTUD5 mRNA in forty-four pairs of HCC tissues and matched tumour-adjacent tissues were detected by RT-qPCR.

Supplementary Figure 2 IHC staining of SLC38A1 in xenografted tumour tissues derived from OTUD5 knockdown and control cells *P < 0.05.

Supplementary Figure 3 OTUD5 does not affect SLC38A1 mRNA expression in HCC cells. (A) HepG2 and Hep3B cells that were transduced with an empty vector (EV) or an OTUD5 expression vector were subjected to RT-qPCR for SLC38A1 and OTUD5 mRNA expression. (B) HCCLM3 and Huh-7 cells that were transduced with OTUD5 shRNAs or NT shRNA were subjected to RT-qPCR for SLC38A1 and OTUD5 mRNA expression. *P < 0.05.

Supplementary Figure 4 OTUD5 deubiquitinates and stabilizes SLC38A1 in HCC cells. (A) Huh7 cells with OTUD5 knockdown were treated with or without MG132. The downregulation of SLC38A1 protein induced by OTUD5 knockdown was reversed by MG132. (B and C) OTUD5 knockdown shortened and OTUD5 overexpression prolonged the half-life of the SLC38A1 protein in HCC cells. (D and E) OTUD5 knockdown promoted but OTUD5 overexpression suppressed the ubiquitination of OTUD5 in HCC cells. *P < 0.05.

## Data Availability

No datasets were generated or analysed during the current study.

## Abbreviations

HCC: hepatocellular carcinoma
DUBs: deubiquitinating enzymes
UCHs: ubiquitin carboxyl-terminal hydrolases
USPs: ubiquitin-specific proteases
OTUs: otubain/ovarian tumor-domain containing proteins
MJDs: Machado-Joseph disease domain superfamily
MCPIPs: monocyte chemotactic protein-induced proteins
JAMMs: JAB1/MPN/MOV34 proteases
MINDY: ubiquitin-containing DUB family
TRAF3: TNF receptor-associated factor 3
RNF186: ring finger protein 186
TCGA: The Cancer Genome Atlas
CPTAC: Clinical Proteomic Tumor Analysis Consortium
SLC38A1: solute carrier family 38 member 1

## References

[1] Sung H, Ferlay J, Siegel RL, Laversanne M, Soerjomataram I, Jemal A, Bray F (2021). Global Cancer statistics 2020: GLOBOCAN estimates of incidence and Mortality Worldwide for 36 cancers in 185 countries. CA Cancer J Clin.

[2] Cao W, Chen HD, Yu YW, Li N, Chen WQ (2021). Changing profiles of cancer burden worldwide and in China: a secondary analysis of the global cancer statistics 2020. Chin Med J (Engl).

[3] Popovic D, Vucic D, Dikic I (2014). Ubiquitination in disease pathogenesis and treatment. Nat Med.

[4] Harrigan JA, Jacq X, Martin NM, Jackson SP (2018). Deubiquitylating enzymes and drug discovery: emerging opportunities. Nat Rev Drug Discov.

[5] Kaushal K, Antao AM, Kim KS, Ramakrishna S (2018). Deubiquitinating enzymes in cancer stem cells: functions and targeted inhibition for cancer therapy. Drug Discov Today.

[6] Schauer NJ, Magin RS, Liu X, Doherty LM, Buhrlage SJ (2020). Advances in discovering deubiquitinating enzyme (DUB) inhibitors. J Med Chem.

[7] Yuan L, Lv Y, Li H, Gao H, Song S, Zhang Y, Xing G, Kong X, Wang L, Li Y (2015). Deubiquitylase OTUD3 regulates PTEN stability and suppresses tumorigenesis. Nat Cell Biol.

[8] Hu H, Brittain GC, Chang JH, Puebla-Osorio N, Jin J, Zal A, Xiao Y, Cheng X, Chang M, Fu YX (2013). OTUD7B controls non-canonical NF-kappaB activation through deubiquitination of TRAF3. Nature.

[9] Zhang Z, Du J, Wang S, Shao L, Jin K, Li F, Wei B, Ding W, Fu P, van Dam H (2019). OTUB2 promotes Cancer Metastasis via Hippo-Independent activation of YAP and TAZ. Mol Cell.

[10] Hou T, Dan W, Liu T, Liu B, Wei Y, Yue C, Que T, Ma B, Lei Y, Wang Z (2022). Deubiquitinase OTUD5 modulates mTORC1 signaling to promote bladder cancer progression. Cell Death Dis.

[11] Li F, Sun Q, Liu K, Zhang L, Lin N, You K, Liu M, Kon N, Tian F, Mao Z (2020). OTUD5 cooperates with TRIM25 in transcriptional regulation and tumor progression via deubiquitination activity. Nat Commun.

[12] Zhang Y, Fan Y, Jing X, Zhao L, Liu T, Wang L, Zhang L, Gu S, Zhao X, Teng Y (2021). OTUD5-mediated deubiquitination of YAP in macrophage promotes M2 phenotype polarization and favors triple-negative breast cancer progression. Cancer Lett.

[13] Cho JH, Kim K, Kim SA, Park S, Park BO, Kim JH, Kim SY, Kwon MJ, Han MH, Lee SB (2021). Deubiquitinase OTUD5 is a positive regulator of mTORC1 and mTORC2 signaling pathways. Cell Death Differ.

[14] Wang Y, Yang L, Chen T, Liu X, Guo Y, Zhu Q, Tong X, Yang W, Xu Q, Huang D, Tu K (2019). A novel lncRNA MCM3AP-AS1 promotes the growth of hepatocellular carcinoma by targeting miR-194-5p/FOXA1 axis. Mol Cancer.

[15] Wang L, Sun L, Liu R, Mo H, Niu Y, Chen T, Wang Y, Han S, Tu K, Liu Q (2021). Long non-coding RNA MAPKAPK5-AS1/PLAGL2/HIF-1alpha signaling loop promotes hepatocellular carcinoma progression. J Exp Clin Cancer Res.

[16] Chen T, Wang L, Chen C, Li R, Zhu N, Liu R, Niu Y, Xiao Z, Liu H, Liu Q, Tu K (2023). HIF-1alpha-activated TMEM237 promotes hepatocellular carcinoma progression via the NPHP1/Pyk2/ERK pathway. Cell Mol Life Sci.

[17] Dewson G, Eichhorn PJA, Komander D. Deubiquitinases in cancer. Nat Rev Cancer. 2023;23:842-62. 10.1038/s41568-023-00633-y 37935888

[18] Kang XY, Zhang J, Tang L, Huang L, Tong J, Fu Q (2020). OTU deubiquitinase 5 inhibits the progression of non-small cell lung cancer via regulating p53 and PDCD5. Chem Biol Drug Des.

[19] Li X, Lu B, Zhang L, Yang J, Cheng Y, Yan D (2022). Mechanism of OTUD5 in non-small cell lung cancer cell proliferation, invasion, and migration. Bosn J Basic Med Sci.

[20] Lou B, Ma G, Yu X, Lv F, Xu F, Sun C, Chen Y (2023). Deubiquitinase OTUD5 promotes hepatitis B virus replication by removing K48-linked ubiquitination of HBV core/precore and upregulates HNF4a expressions by inhibiting the ERK1/2/mitogen-activated protein kinase pathway. Cell Mol Life Sci.

[21] Vogel A, Meyer T, Sapisochin G, Salem R, Saborowski A (2022). Hepatocellular carcinoma. Lancet.

[22] Kayagaki N, Phung Q, Chan S, Chaudhari R, Quan C, O’Rourke KM, Eby M, Pietras E, Cheng G, Bazan JF (2007). DUBA: a deubiquitinase that regulates type I interferon production. Science.

[23] Wise DR, Thompson CB (2010). Glutamine addiction: a new therapeutic target in cancer. Trends Biochem Sci.

[24] Wang K, Cao F, Fang W, Hu Y, Chen Y, Ding H, Yu G (2013). Activation of SNAT1/SLC38A1 in human breast cancer: correlation with p-Akt overexpression. BMC Cancer.

[25] Liu Y, Yang Y, Jiang L, Xu H, Wei J. High Expression Levels of SLC38A1 Are Correlated with Poor Prognosis and Defective Immune Infiltration in Hepatocellular Carcinoma. *J Oncol* 2021, 2021:5680968. 10.1155/2021/5680968 PMC8541878 34697542

[26] Feng HG, Wu CX, Zhong GC, Gong JP, Miao CM, Xiong B (2023). Integrative analysis reveals that SLC38A1 promotes hepatocellular carcinoma development via PI3K/AKT/mTOR signaling via glutamine mediated energy metabolism. J Cancer Res Clin Oncol.

[27] Xie J, Li P, Gao HF, Qian JX, Yuan LY, Wang JJ (2014). Overexpression of SLC38A1 is associated with poorer prognosis in Chinese patients with gastric cancer. BMC Gastroenterol.

[28] Zhou FF, Xie W, Chen SQ, Wang XK, Liu Q, Pan XK, Su F, Feng MH (2017). SLC38A1 promotes proliferation and migration of human colorectal cancer cells. J Huazhong Univ Sci Technolog Med Sci.

[29] Park YY, Sohn BH, Johnson RL, Kang MH, Kim SB, Shim JJ, Mangala LS, Kim JH, Yoo JE, Rodriguez-Aguayo C (2016). Yes-associated protein 1 and transcriptional coactivator with PDZ-binding motif activate the mammalian target of rapamycin complex 1 pathway by regulating amino acid transporters in hepatocellular carcinoma. Hepatology.

