# Supplementary material for: OTUD5 promotes the growth of hepatocellular carcinoma by deubiquitinating and stabilizing SLC38A1
Source: Biol Direct. 2024 Apr 24;19:31. doi: 10.1186/s13062-024-00475-0 (PMC11041014; doi:10.1186/s13062-024-00475-0)
Supplement: Supplementary file 1 — Supplementary Material 1 [file 13062_2024_475_MOESM1_ESM.docx]

**Supplementary Table 1 The sequence of shRNAs used in this study**

| **ID** | **5’** | **stem** | **loop** | **stem** | **3’** |
| --- | --- | --- | --- | --- | --- |
| OTUD5-1  sense | Ccgg | CCATCATTCAAACCAGGGTTT | CTCGAG | AAACCCTGGTTTGAATGATGG | TTTTTg |
| OTUD5-1 antisense | aattcaaaaa | CCATCATTCAAACCAGGGTTT | CTCGAG | AAACCCTGGTTTGAATGATGG |  |
| OTUD5-2 sense | Ccgg | CCGACTACTTCTCCAACTATG | CTCGAG | CATAGTTGGAGAAGTAGTCGG | TTTTTg |
| OTUD5-2 antisense | aattcaaaaa | CCGACTACTTCTCCAACTATG | CTCGAG | CATAGTTGGAGAAGTAGTCGG |  |
| SLC38A1 sense | Ccgg | GGTGGTGATAGTTACCTTTGG | CTCGAG | CCAAAGGTAACTATCACCACC | TTTTTg |
| SLC38A1 antisese | aattcaaaaa | GGTGGTGATAGTTACCTTTGG | CTCGAG | CCAAAGGTAACTATCACCACC |  |
