# Supplementary figures and images for: OTUD5 promotes the growth of hepatocellular carcinoma by deubiquitinating and stabilizing SLC38A1
Source: Biol Direct. 2024 Apr 24;19:31. doi: 10.1186/s13062-024-00475-0 (PMC11041014; doi:10.1186/s13062-024-00475-0)

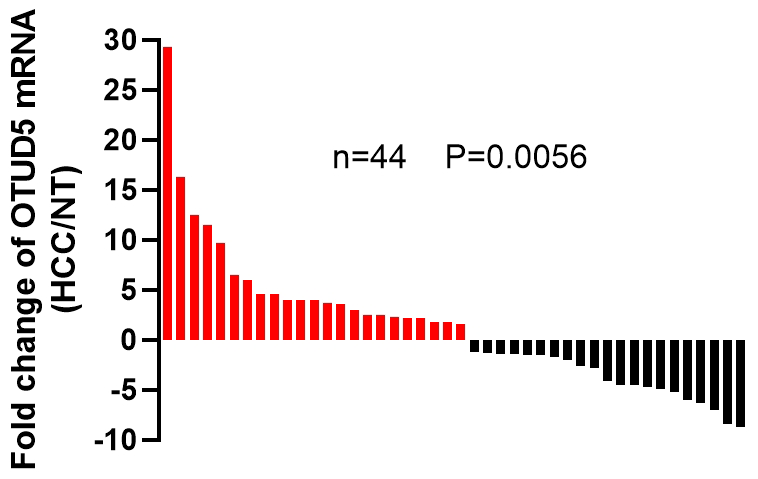

Supplement: Supplementary file 3 — Supplementary Figure 1 The expression of OTUD5 mRNA in forty-four pairs of HCC tissues and matched tumour-adjacent tissues were detected by RT-qPCR. [file 13062_2024_475_MOESM3_ESM.tif]

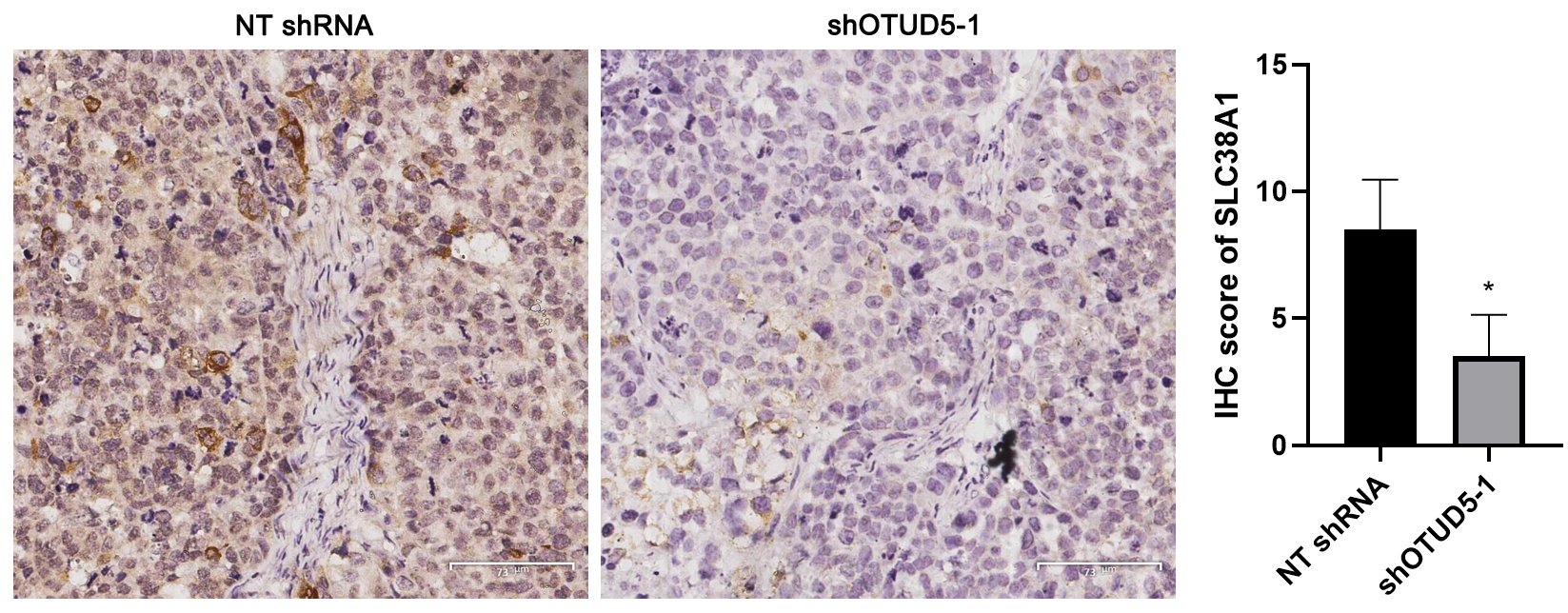

Supplement: Supplementary file 4 — Supplementary Figure 2 IHC staining of SLC38A1 in xenografted tumour tissues derived from OTUD5 knockdown and control cells *P < 0.05. [file 13062_2024_475_MOESM4_ESM.tif]

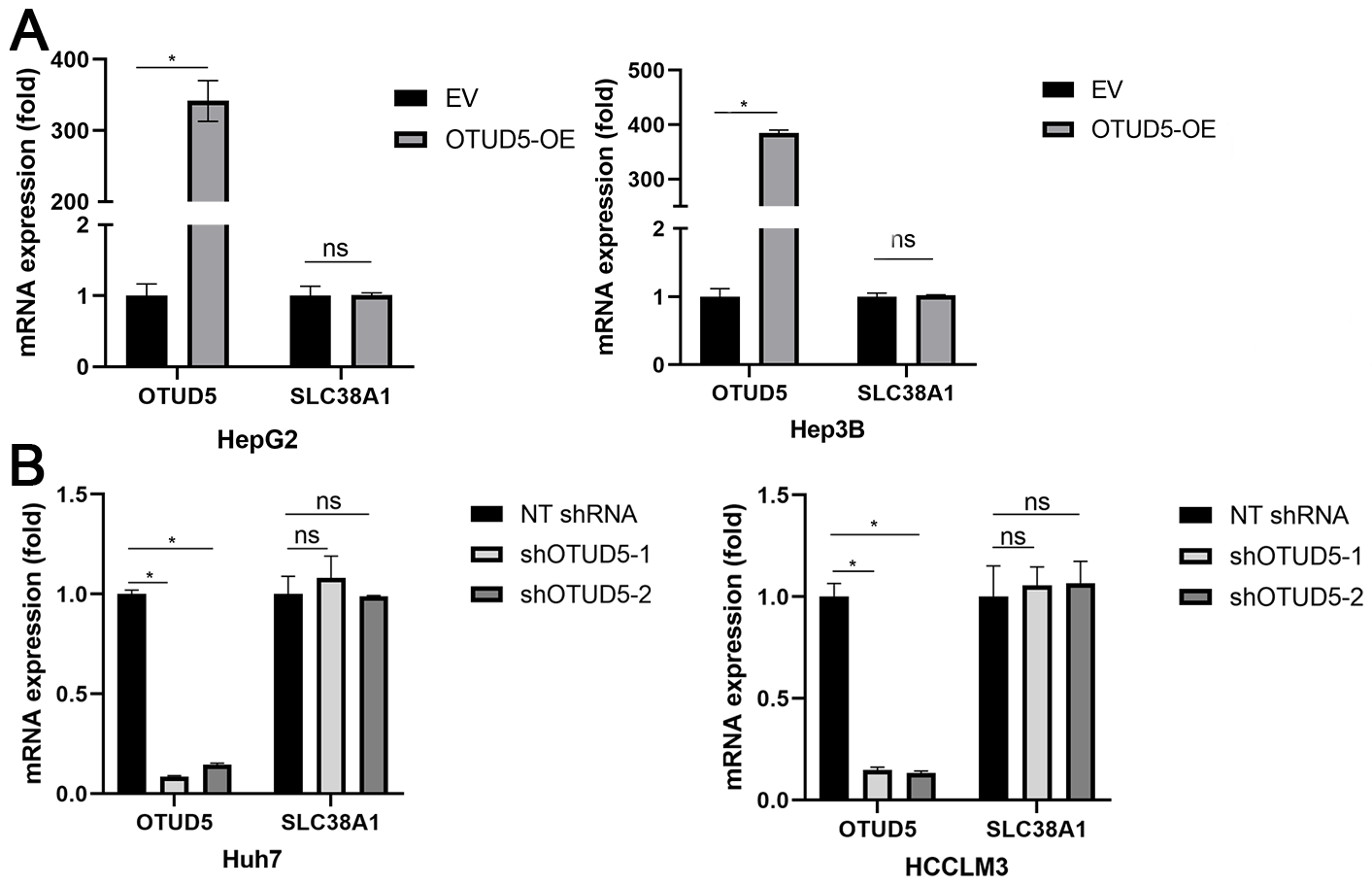

Supplement: Supplementary file 5 — Supplementary Figure 3 OTUD5 does not affect SLC38A1 mRNA expression in HCC cells. (A) HepG2 and Hep3B cells that were transduced with an empty vector (EV) or an OTUD5 expression vector were subjected to RT-qPCR for SLC38A1 and OTUD5 mRNA expression. (B) HCCLM3 and Huh-7 cells that were transduced with OTUD5 shRNAs or NT shRNA were subjected to RT-qPCR for SLC38A1 and OTUD5 mRNA expression. *P < 0.05. [file 13062_2024_475_MOESM5_ESM.tif]

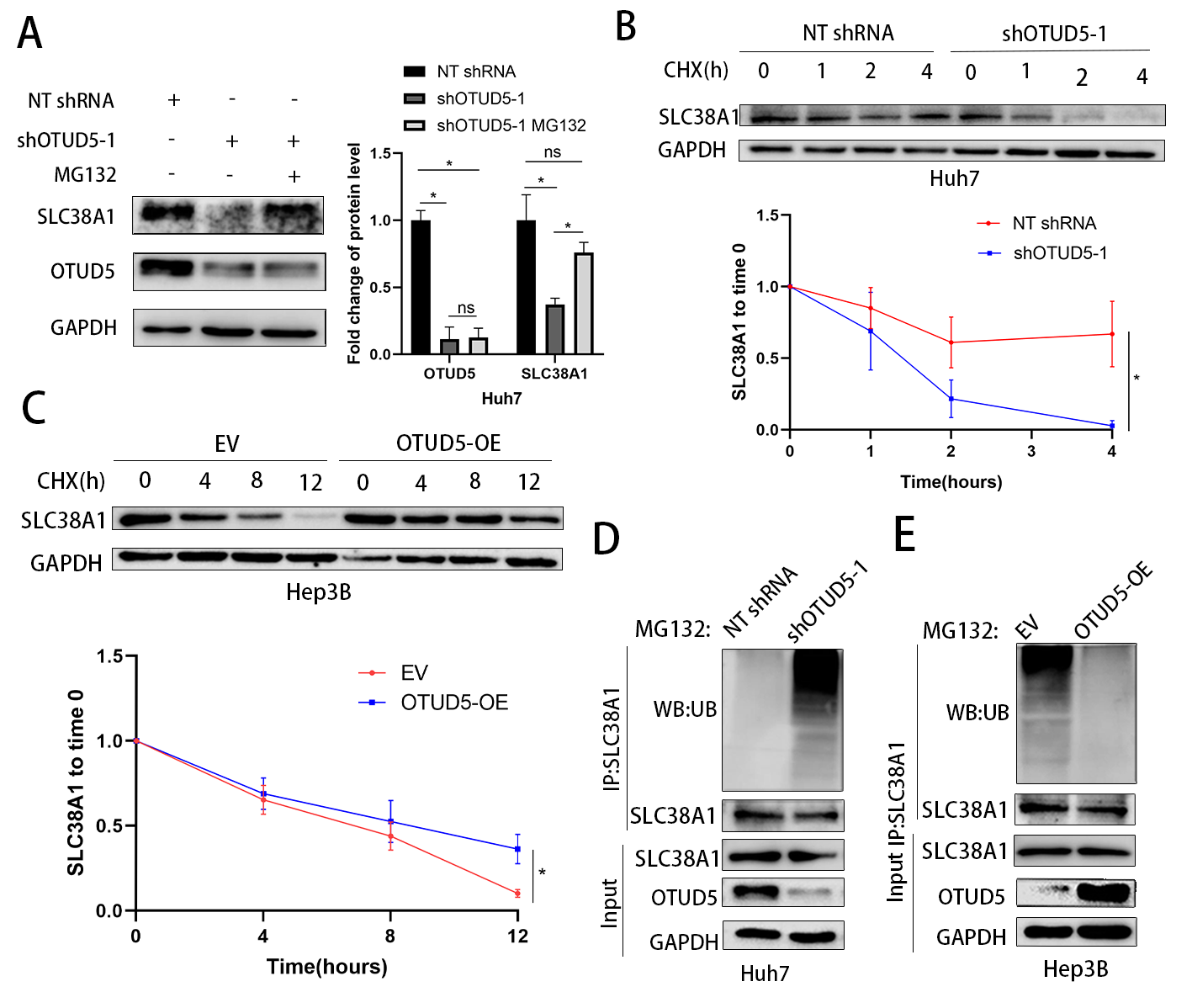

Supplement: Supplementary file 6 — Supplementary Figure 4 OTUD5 deubiquitinates and stabilizes SLC38A1 in HCC cells. (A) Huh7 cells with OTUD5 knockdown were treated with or without MG132. The downregulation of SLC38A1 protein induced by OTUD5 knockdown was reversed by MG132. (B and C) OTUD5 knockdown shortened and OTUD5 overexpression prolonged the half-life of the SLC38A1 protein in HCC cells. (D and E) OTUD5 knockdown promoted but OTUD5 overexpression suppressed the ubiquitination of OTUD5 in HCC cells. *P < 0.05. [file 13062_2024_475_MOESM6_ESM.tif]
